# The effect of the macrolide antibiotic tylosin on microbial diversity in the canine small intestine as demonstrated by massive parallel 16S rRNA gene sequencing

**DOI:** 10.1186/1471-2180-9-210

**Published:** 2009-10-02

**Authors:** Jan S Suchodolski, Scot E Dowd, Elias Westermarck, Jörg M Steiner, Randy D Wolcott, Thomas Spillmann, Jaana A Harmoinen

**Affiliations:** 1 Gastrointestinal Laboratory, Texas A&M University, College Station, TX, USA; 2 Research and Testing Laboratory and Medical Biofilm Research Institute Lubbock, TX, USA; 3 Department of Clinical Veterinary Sciences, Helsinki University, Helsinki, Finland

## Abstract

**Background:**

Recent studies have shown that the fecal microbiota is generally resilient to short-term antibiotic administration, but some bacterial taxa may remain depressed for several months. Limited information is available about the effect of antimicrobials on small intestinal microbiota, an important contributor to gastrointestinal health. The antibiotic tylosin is often successfully used for the treatment of chronic diarrhea in dogs, but its exact mode of action and its effect on the intestinal microbiota remain unknown. The aim of this study was to evaluate the effect of tylosin on canine jejunal microbiota. Tylosin was administered at 20 to 22 mg/kg q 24 hr for 14 days to five healthy dogs, each with a pre-existing jejunal fistula. Jejunal brush samples were collected through the fistula on days 0, 14, and 28 (14 days after withdrawal of tylosin). Bacterial diversity was characterized using massive parallel 16S rRNA gene pyrosequencing.

**Results:**

Pyrosequencing revealed a previously unrecognized species richness in the canine small intestine. Ten bacterial phyla were identified. Microbial populations were phylogenetically more similar during tylosin treatment. However, a remarkable inter-individual response was observed for specific taxa. *Fusobacteria, Bacteroidales*, and *Moraxella *tended to decrease. The proportions of *Enterococcus*-like organisms, *Pasteurella *spp., and *Dietzia *spp. increased significantly during tylosin administration (p < 0.05). The proportion of *Escherichia coli-*like organisms increased by day 28 (p = 0.04). These changes were not accompanied by any obvious clinical effects. On day 28, the phylogenetic composition of the microbiota was similar to day 0 in only 2 of 5 dogs. Bacterial diversity resembled the pre-treatment state in 3 of 5 dogs. Several bacterial taxa such as *Spirochaetes*, *Streptomycetaceae*, and *Prevotellaceae *failed to recover at day 28 (p < 0.05). Several bacterial groups considered to be sensitive to tylosin increased in their proportions.

**Conclusion:**

Tylosin may lead to prolonged effects on the composition and diversity of jejunal microbiota. However, these changes were not associated with any short-term clinical signs of gastrointestinal disease in healthy dogs. Our results illustrate the complexity of the intestinal microbiota and the challenges associated with evaluating the effect of antibiotic administration on the various bacterial groups and their potential interactions.

## Background

The gastrointestinal tract of humans and animals is inhabitated by a specialized microbiota, but our understanding of the composition and the dynamics of this intestinal ecosystem is very rudimentary. Recent molecular methodologies, typically based on amplification and identification of 16S ribosomal RNA genes, have revealed highly complex and diverse bacterial, fungal, and viral communities within the intestinal tract of mammals [1-4].

The composition of the intestinal microbial ecosystem has a significant impact on the health status of an individual. The intestinal microbiota are a key player in the development of the host immune system, provide trophic metabolites and energy to the host, and also aid in the resistance against colonization of pathogens [5]. At the same time, derangements of the intestinal microbiota or the invasion with specific pathogens have been implicated as a cause for gastrointestinal disease [6,7].

Nutritional or medical intervention, especially the use of antimicrobials can lead to general alterations in intestinal microbiota [8,9]. Tylosin, a member of the macrolide class of antibiotics, is commonly recommended for the treatment of chronic enteropathies in dogs. It is currently unknown if tylosin at therapeutic doses has a direct effect on intestinal pathogens or if it leads to a more general modulation of the intestinal microbiota in dogs with diarrhea, with a subsequent improvement of intestinal digestion and absorption. For example, some known gastrointestinal pathogens, including *Clostridium perfringens *and *Campylobacter *spp., are known to play a role in the etiopathogenesis of chronic or intermittent diarrhea in dogs, and these bacteria are generally sensitive to tylosin [10]. Tylosin is also a commonly used antibiotic for the treatment of canine small intestinal bacterial overgrowth (SIBO) or antibiotic responsive diarrhea (ARD) [11]. Recently the term tylosin-responsive diarrhea has been introduced, because tylosin treatment led to the best therapeutic response in a subpopulation of dogs with chronic diarrhea [12]. Tylosin-responsive diarrhea (TRD) affects typically middle-aged, large-breed dogs and clinical signs indicate that TRD affects both the small and large intestine. The etiology of TRD is currently unknown. Diarrhea usually improves within a few days, but often recurs within a few weeks after cessation of tylosin administration and the majority of dogs require lifelong therapy [12]. However, in addition to its antimicrobial effect, a direct anti-inflammatory effect of tylosin has also been proposed. This anti-inflammatory effect has been speculated to be due to the modulation of cyclooxygenase-2, nitric oxidase synthase, and several cytokines [13]. In mice and Rhesus Macaques with colitis, tylosin has also been shown to reduce macroscopic lesion scores, and either a direct immunomodulatory effect or an indirect effect due to the modulation of the microbiota has been suggested [14,15].

Antibiotic activity has a profound effect on the intestinal microbiota [8,16], and it is important to characterize changes in bacterial diversity, their magnitude and the resilience of the intestinal microbiota against antibiotic-related modifications. Such an understanding could potentially lead to the development of alternative treatment modalities that would allow therapeutic options other than the use of antimicrobials. While recent studies have shown that the fecal microbiota is generally resilient to short-term antibiotic administration, some bacterial taxa may remain depressed for several months [8,16]. Limited information concerning the effect of antimicrobials on small intestinal microbiota, an important contributor to gastrointestinal health, is available. Previous studies have examined the effect of tylosin on intestinal microbiota in pigs and chickens using culture based methods or molecular fingerprinting tools, but detailed sequencing data have not been provided [17,18]. Therefore, the aim of this study was to evaluate the effect of tylosin on the jejunal microbiota by massive parallel 16S rRNA gene pyrosequencing.

In this study we administered tylosin at therapeutic doses to healthy dogs with a pre-existing jejunal fistula and analyzed changes in bacterial communities before, during, and 14 days after cessation of tylosin by 16S rRNA gene pyrosequencing. Our results indicate a previously uncharacterized high species richness in the canine jejunum. Tylosin had a profound effect on the microbial composition in the small intestine of dogs. Furthermore, tylosin had also a pervasive effect on specific bacterial taxa, which failed to recover within 14 days. However, these changes were not associated with any short-term clinical signs of gastrointestinal disease in healthy dogs. Our results illustrate the complexity of the intestinal microbiota and the challenges associated with evaluating the effect of antibiotic administration on the various bacterial groups and their potential interactions. The results also suggest that the proposed mode of action of an antibiotic on different bacterial genera does not necessarily match the *in vivo *effects, as several bacterial groups that are considered to be sensitive to tylosin increased in their proportions.

## Results

### Animals

All dogs tolerated the course of antibiotics well and no obvious side effects (e.g., clinical signs of gastrointestinal disease such as diarrhea) were noted during the study period. The body weights or body condition scores of the dogs did not change during the study.

### Characterization of the canine small intestinal microbiota

**Figure 1:**
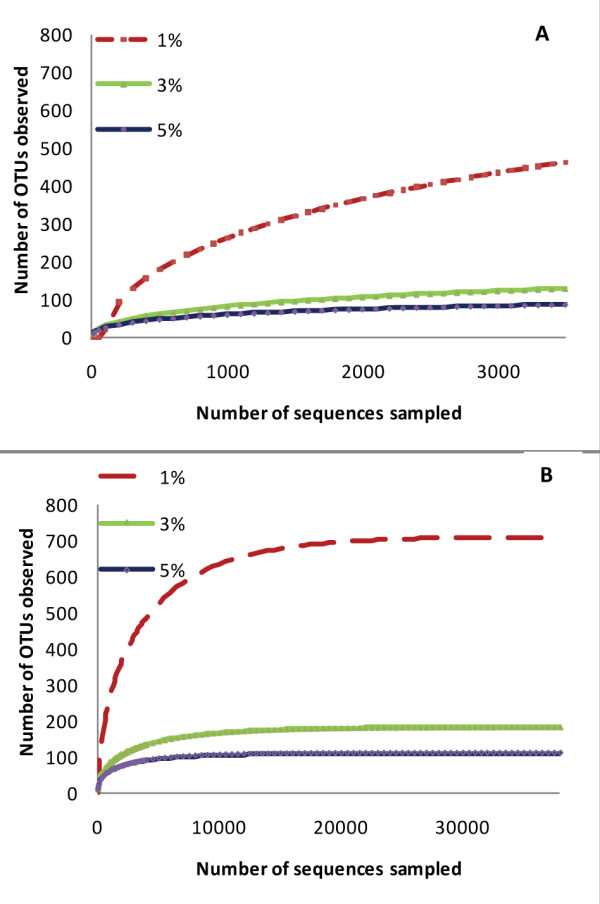
**Representative rarefaction curves depicting the effect of 1%, 3%, and 5% dissimilarity on the number of identified and maximum predicted operative taxonomical units (OTUs) in one dog**. (A) This plot shows that with the average number of collected sequencing tags per dog (mean ± SD: 3188 ± 1091 sequencing tags), we underestimated the number of OTUs at 1% dissimilarity. A reasonable coverage was obtained at 3% and 5% dissimilarity (curves approximate a parallel line to the x-axis). (B) To estimate the maximum number of OTUs at various dissimilarities, a Richards equation was fit to the rarefaction curves. The results indicate that approximately 38,000 sequences would need to be sampled to cover 100% of the expected OTUs in the canine jejunum.

A total of 44,069 pyrosequencing tags were evaluated across all 15 samples (mean ± SD: 3188 ± 1091 sequencing tags per sample). All dogs showed highly diverse microbial communities within their small intestine. Table 1 lists the mean number of obtained and maximum predicted OTUs and richness estimators at strain (1% dissimilarity), species (3%), and genus (5%) level [19]. At day 0 and at 3% dissimilarity, which is commonly used to describe the species level [19], a range of 25-453 OTUs (mean: 218 OTUs) was observed, indicating strong inter-individual differences in microbial diversity in the canine jejunum. The Chao 1 and Ace richness estimators were used to estimate the total number of OTUs in the canine jejunum. On day 0 and at 3% dissimilarity, the Chao 1 estimated between 32 and 707 OTUs (mean: 342 OTUs) per sample, and the Ace estimated between 32 and 721 OTUs (mean: 332 OTUs) per sample. To estimate the maximum number of OTUs at various dissimilarities, a Richards equation was fit to the obtained rarefaction curves [20]. Table 1 shows the mean number of maximum predicted OTUs in the canine jejunum: on day 0 (begin of the study) and at 3% dissimilarity (species level), the maximum predicted number of OTUs ranged from 32 to 666 (mean: 293 OTUs). At 1% dissimilarity (strain level), a mean of 950 OTUs (range: 183 to 1,789) was predicted. Figure 1 illustrates that with the average number of sequencing tags collected per dog in this study (mean ± SD: 3188 ± 1091 sequencing tags), we underestimated the maximum number of OTUs at 1% dissimilarity. However, at 3% and 5% dissimilarity the rarefaction curves approximate a parallel line to the x-axis, suggesting that a reasonable coverage was obtained at the species and genus level. Using the Richard's equation we calculated that approximately 38,000 sequences would need to be sampled to identify 100% of the expected OTUs in the canine jejunum (Figure 1B). To obtain a complete coverage at 0% dissimilarity, approx. 106,000 sequences would need to be analyzed (data not shown).

**Table 1:** Mean values for various indices.

	**Shannon-Weaver index**			**OTU**			**maximum predicted OTU**		
			
	**1%**	**3%**	**5%**	**1%**	**3%**	**5%**	**1%**	**3%**	**5%**
			
**day 0**	4.55	2.88	2.03	695	218	143	950	293	169
**day 14**	4.58	2.84	1.87	594	149	93	789	197	111
**day 28**	3.98	2.60	1.46	542	115	72	637	136	90
									
	**Rarefaction**			**Chao 1**			**ACE**		
			
	**1%**	**3%**	**5%**	**1%**	**3%**	**5%**	**1%**	**3%**	**5%**
			
**day 0**	690	217	142	984	342	197	1030	332	191
**day 14**	590	148	92	794	204	123	807	209	124
**day 28**	539	115	72	669	150	86	660	155	92

This table shows the Shannon-Weaver bacterial diversity index, observed operative taxonomical units (OTU), the predicted maximum number of OTUs in the canine jejunum, rarefaction, and species richness estimators (ACE and Chao 1) at strain (1% dissimilarity), species (3%), and genus (5%) level across the three sampling periods. Tylosin administration led to a progressive decrease in mean indices, which were lowest on day 28 (14 days after cessation of tylosin). However, a strong individual variation was observed among all dogs (see text).

**Figure 2:**
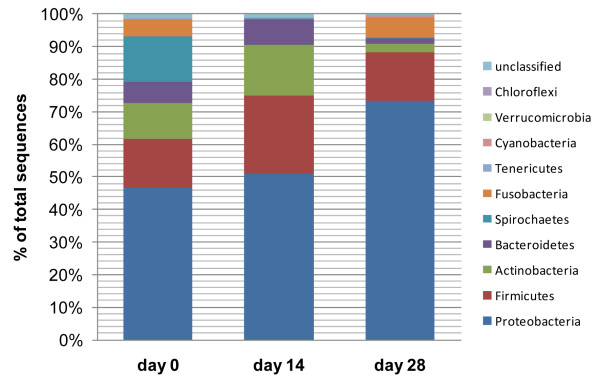
**Distributions of major bacterial groups at the phylum level**. (day 0 = baseline; day 14 = after 14 days of tylosin administration; day 28 = 2 weeks after cessation of tylosin therapy).

On day 0, ten different bacterial phyla were identified. The major bacterial phyla were *Proteobacteria *(46.7% of all sequences), *Firmicutes *(15.0%), *Actinobacteria *(11.2%), *Spirochaetes *(14.2%), *Bacteroidetes *(6.2%), and *Fusobacteria *(5.4%). The phyla *Tenericutes, Verrucomicrobia, Cyanobacteria*, and *Chloroflexi *accounted for < 0.1% of all obtained sequencing tags each (Figure 2).

### Effect of tylosin on diversity indices and species richness estimators

**Figure 3:**
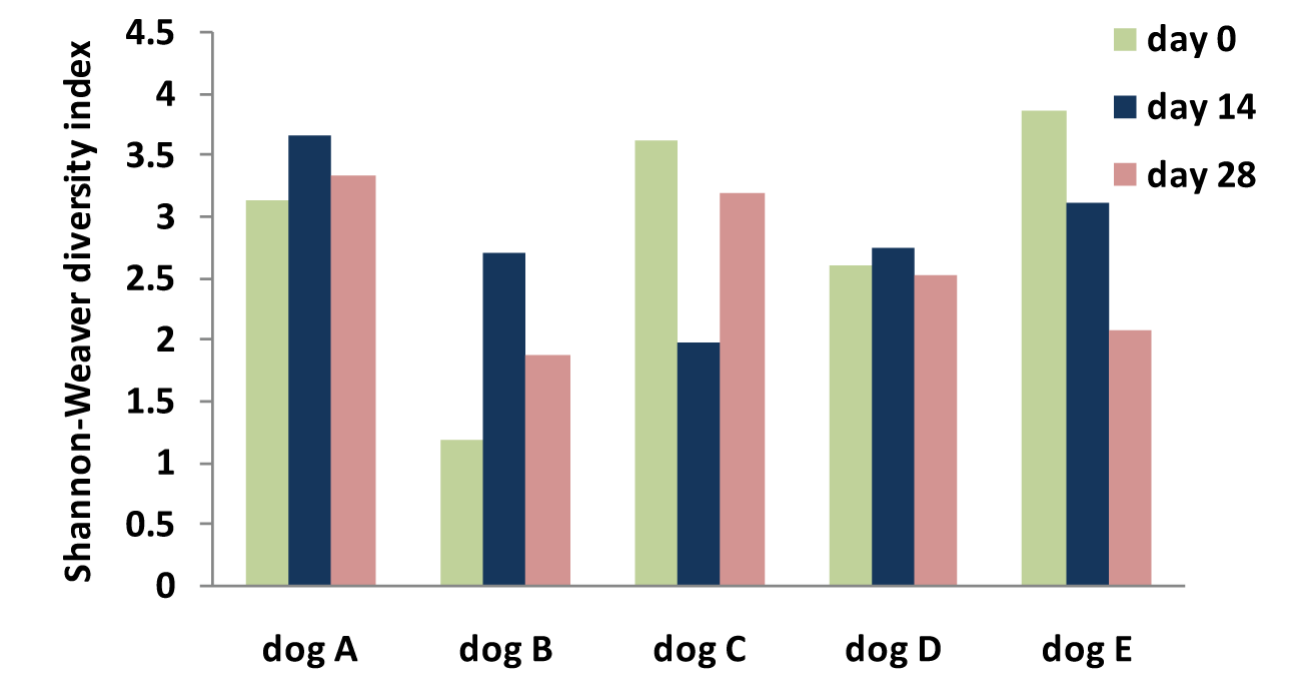
**Shannon-Weaver bacterial diversity index across the 3 sampling periods for the 5 individual dogs**. A strong individual response in bacterial diversity to tylosin treatment was observed in all dogs. (day 0 = baseline; day 14 = after 14 days of tylosin administration; day 28 = 2 weeks after cessation of tylosin therapy).

While tylosin administration led to a progressive decrease in mean bacterial diversity and species richness estimators over the three sampling periods (Table 1), this effect was not consistent for all dogs. In fact, on day 14 (i.e., samples collected at the end of tylosin administration) the Shannon-Weaver diversity index increased moderately in 2 dogs and markedly in 1 dog (Figure 3). Similar results were obtained for OTUs and the Chao 1 and Ace estimators. On day 28 (14 days after cessation of tylosin administration), the diversity indices and richness estimators were markedly decreased in 2 out of 5 dogs when compared to baseline.

### Effect of tylosin on small intestinal microbial communities

**Figure 4:**
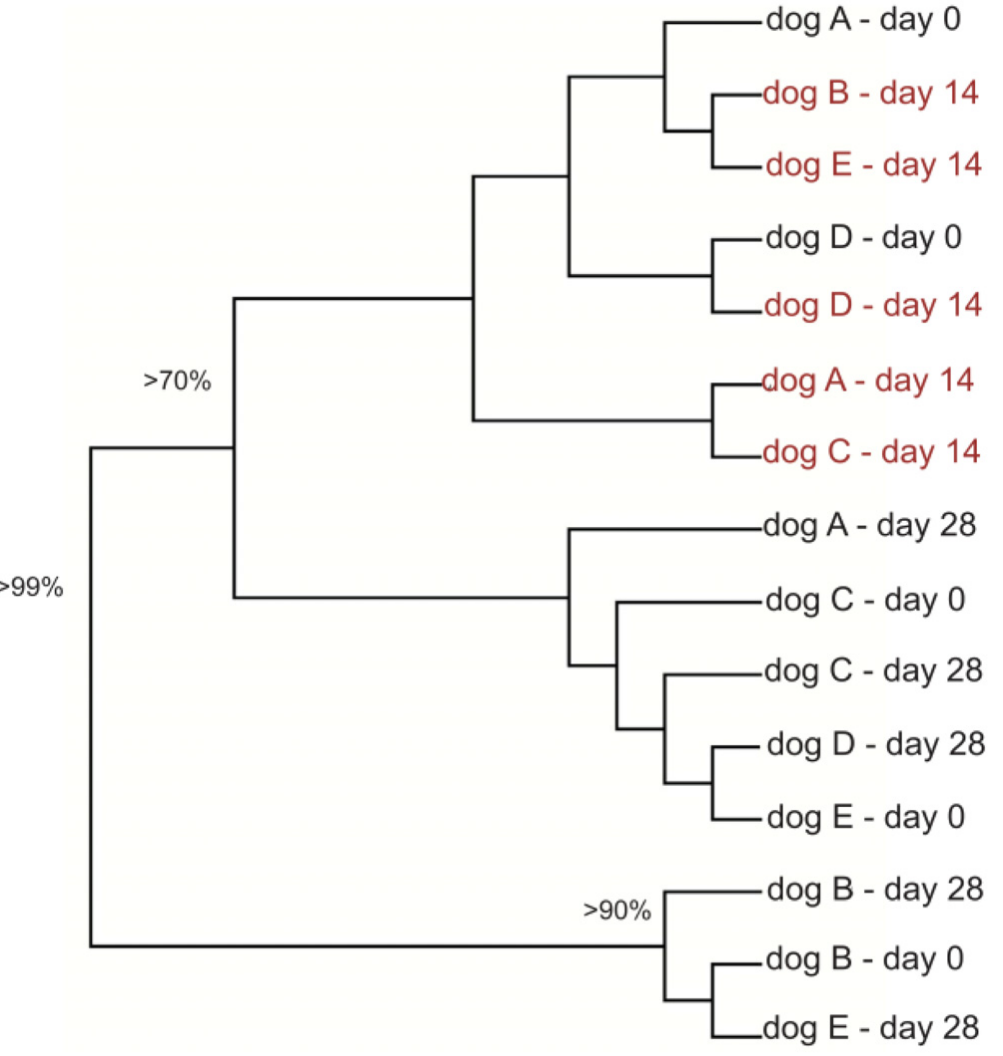
**Dendrogram illustrating the phylogenetic clustering of the microbiota in all 5 dogs enrolled in this study across the 3 sampling periods**. The dendrogram was constructed using the unweight UniFrac distance metric. The numbers at the nodes indicate Jackknife values (i.e., number of times the node was recovered after 100 replicates). Jackknife values < 50% are not shown. This dendrogram illustrates that the samples obtained after 14 days of tylosin administration (day 14, in red) tended to form a cluster (Jackknife value > 70%).

**Figure 5:**
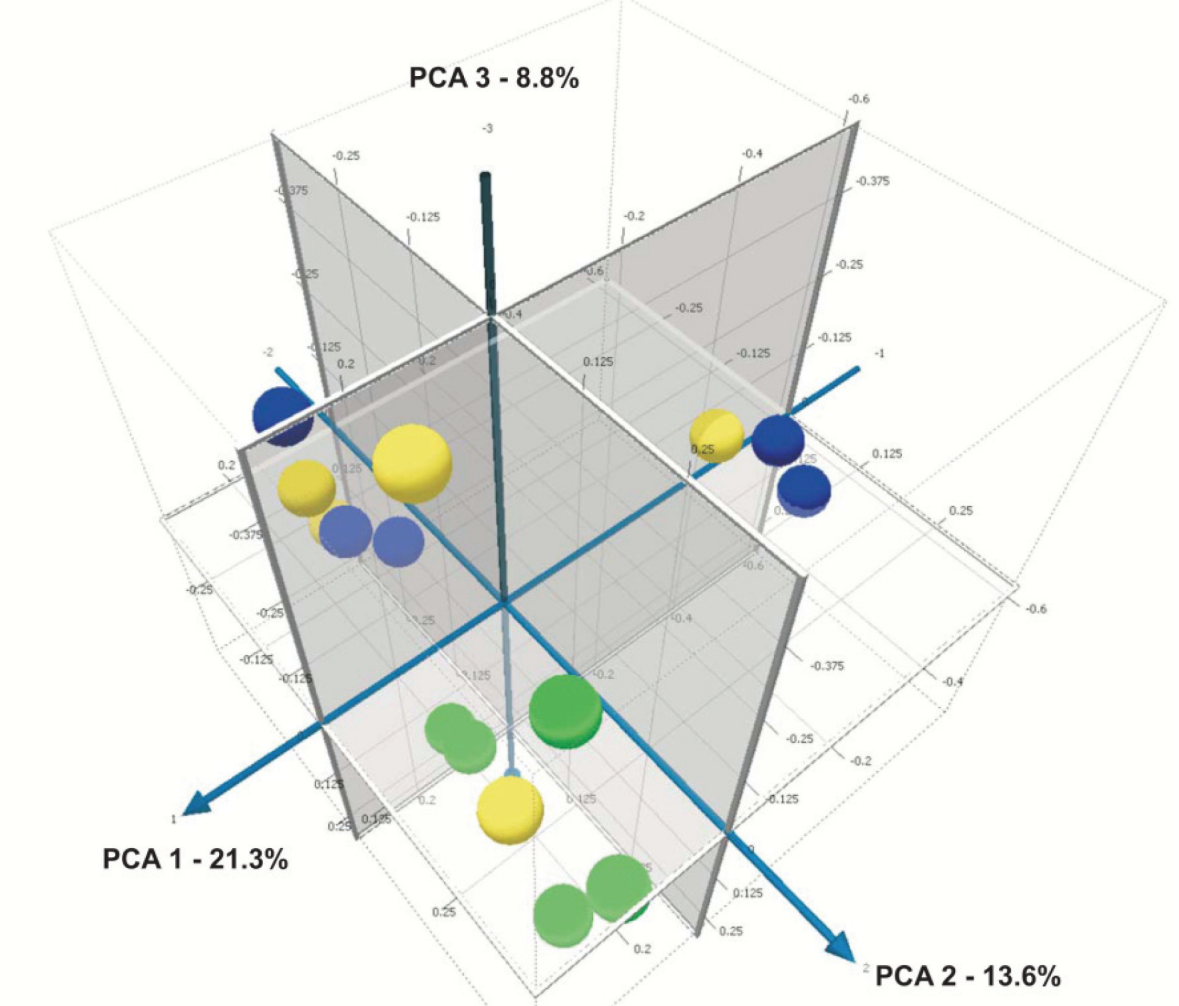
**Principal Component Analysis (PCA) plot generated using the unweighted (based on the presence or absence of different taxa without regard to abundance) UniFrac distance metric**. illustrating the phylogenetic relationship of microbial communities in all dogs at the 3 treatment periods (yellow = day 0; green = after 14 days of tylosin treatment; blue = day 28, 2 weeks after cessation of tylosin treatment). Tylosin associated samples (green, day 14) were separated from the non tylosin associated samples mostly along PCA axis 2 (accounting for 13.5% of all variability between samples), indicating that tylosin treatment had an effect on the microbial composition of the jejunal microbiota.

Results of the UniFrac distance metric indicated that tylosin led to a significant shift in microbial populations (p < 0.05). Microbial communities tended to form a cluster during tylosin treatment (Figure 4). A PCA plot was generated using the unweighted UniFrac distance metric, which takes into account the presence or absence of different taxa without regard to their abundance (Figure 5). Tylosin associated samples (green, day 14) were separated from the non tylosin associated samples mostly along PCA axis 2 (accounting for 13.5% of all variability between samples). On day 28, the phylogenetic composition of the microbiota was similar to day 0 in only 2 of 5 dogs (Figure 4). Bacterial diversity as measured by the Shannon-Weaver diversity index resembled the pre-treatment state in 3 of 5 dogs (Figure 3). Several bacterial groups changed in their proportions in response to tylosin, but a high inter-individual response was observed for various bacterial taxa. Proportions of *Spirochaetes, Fusobacteria, Bacteroidales, Moraxella*, and *Bacilli *tended to decrease during tylosin administration.

### Spirochaetes

*Spirochaetes *were found in all 5 dogs at baseline (mean: 14.15%, range: 0.05% to 62.97% of all identified sequences). On day 14, sequences of *Spirochaetes *were found in 2 of 5 dogs, with a reduction of the mean to 0.02% (range 0.00% to 0.06%; p = 0.039). This bacterial phylum was found on day 28 only in 3 of 5 dogs (mean 0.36%, range 0.00% to 1.48%). In the dog with the highest proportion of sequences belonging to *Spirochaetes *at baseline (62.97%), no such sequences were identified on days 14 or 28.

### Fusobacteria

*Fusobacteria *were detected in 3 of 5 dogs at baseline, but this bacterial phylum was a major constituent of the jejunal microbiota in only 1 dog (18.22% of all sequences). In this dog, *Fusobacteria *decreased to 0.16% on day 14, and rebounded to 27.98% on day 28. In the remaining dogs, *Fusobacteria *were detected at low proportions (range 0.00% to 2.25%) at the three sampling points, and overall no significant changes were observed for this phylum.

### Bacteroidetes

Sequences belonging to the phylum *Bacteroidetes *were detected in all dogs at all 3 time points (mean 5.34% of all sequences). This group showed marked inter-individual differences in the response to tylosin on the phylum level. On day 14 the proportions of *Bacteroidetes *were increased in 3 dogs, decreased in 1 dog, and unchanged in 1 dog. On day 28, there was a trend for the proportions of *Bacteroidetes *to return to baseline values. Analysis on various phylogenetic levels revealed that the proportions of *Flavobacteriacae *increased by day 14 (marked increase in 3 of 5 dogs) and returned to baseline by day 28 (p = 0.09). In contrast, the order *Bacteroidales *decreased in proportions in all 5 dogs by day 14 (mean 5.95% on day 0 vs. 0.12% on day 14), and tended to return to baseline by day 28 (mean 1.63% on day 28; p = 0.09). This was predominantly due to a significant decrease in *Prevotellaceae *(mean 2.09% on day 0 vs. 0.03% on day 14; p = 0.039). Furthermore, *Prevotellaceae *did not recover by day 28 and were not detected in any of the dogs at this time point. *Bacteroidaceae *decreased by day 14 (mean 1.71% on day 0 vs. 0.06% on day 14), but this effect was not significant (p = 0.49). Furthermore, *Bacteroidaceae *increased by day 28 (mean 0.42% of all sequences).

### Firmicutes

**Figure 6:**
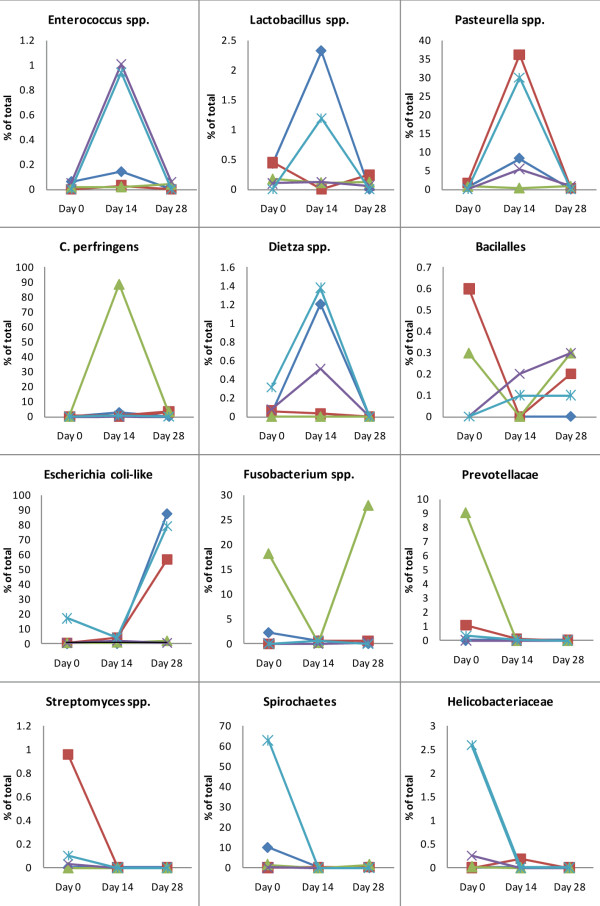
**Responses of specific bacterial groups to tylosin treatment**. Each dog is represented by the same symbol and color across all panels. (dog A: red square, dog B: light blue asterisk, dog C: green triangle, dog D: purple X, dog E: dark blue diamond). The numbering of all dogs is the same as in Figures 3, 4 and 8. (Note: scale of y-axis differs between panels).

The phylum *Firmicutes *was the second most abundant bacterial group in the canine jejunum (Figure 2). On a phylum level, no significant changes were observed across the three time points for *Firmicutes. Clostridiaceae *increased from 5.47% to 19.46% and decreased to 10.72% by day 28. However, this data was skewed due to a marked shift of the microbiota observed in one dog, where sequences belonging to *C. perfringens*-like organisms increased from 21.8% to 86.47% to 33.6% across the three time points (Figure 6). In the remaining dogs, *Clostridium *spp. showed only moderate changes by day 14 and 28, and overall no significant changes were observed for this bacterial group (p = 0.52).

Inter-individual differences were observed for *Bacillales*, and their proportions increased in 2 dogs and decreased in 3 dogs by day 14 (Figure 6). *Lactobacillales *decreased in 4 dogs, but increased in 1 dog by day 14, and tended to return to baseline values by day 28 (p = 0.12). On a genus level, inter-individual differences were observed for *Lactobacillus*-like organisms, which increased in 2 dogs, remained stable in 2, and decreased in 1 dog by day 14, and tended to return to baseline values by day 28 (p = 0.36). The proportions of *Enterococcus*-like organisms increased from 0.3% to 1.1% to 0.1% by day 28 (p < 0.01) (Figure 6). This increase was observed in 4 of 5 dogs, whereas the proportions remained stable in the remaining dog.

### Proteobacteria

**Figure 7:**
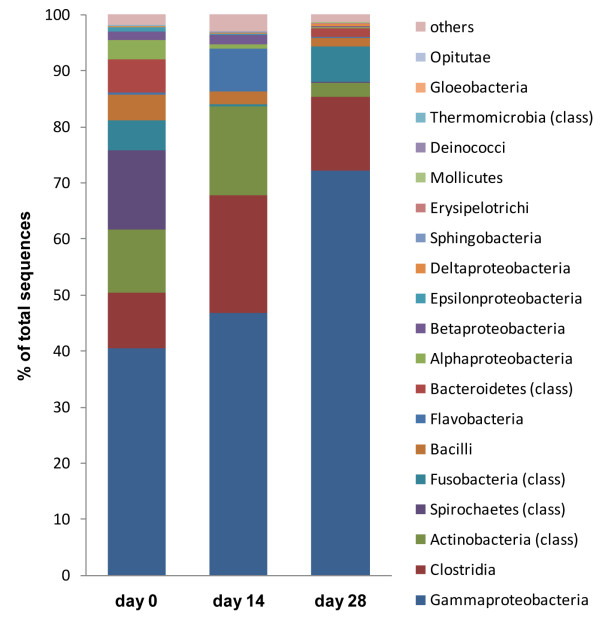
**Distribution of major bacterial groups on a class level**. (day 0 = baseline; day 14 = after 14 days of tylosin administration; day 28 = 2 weeks after cessation of tylosin therapy).

**Figure 8:**
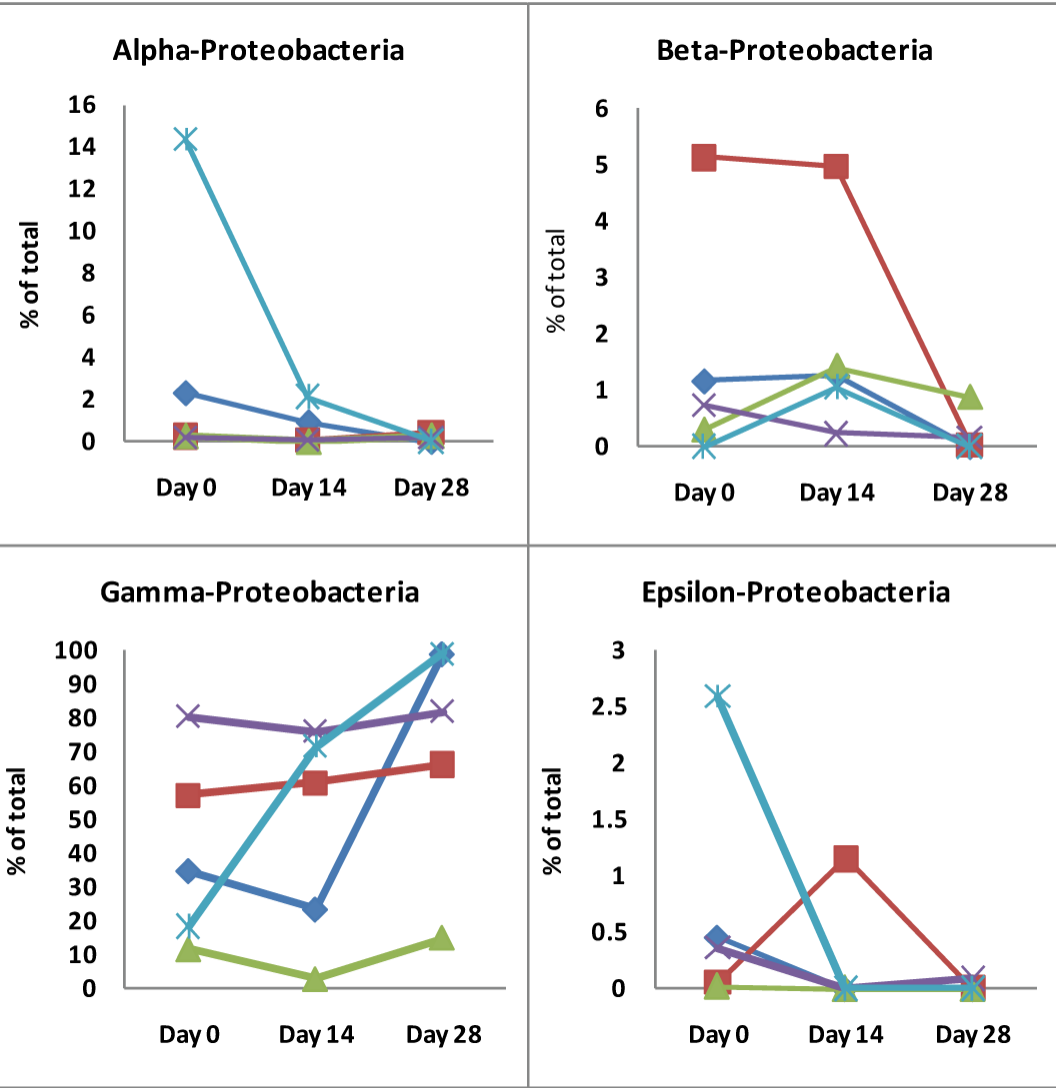
**Changes in the sequences identified, belonging to the different classes of α, β, γ, and ε-*Proteobacteria***. Each dog is represented by the same symbol and color across all panels. (dog A: red square, dog B: light blue asterisk, dog C: green triangle, dog D: purple X, dog E: dark blue diamond). The numbering of all dogs is the same as in Figures 3, 4 and 6. (Note: scale of y-axis differs between panels).

The phylum *Proteobacteria *was the most abundant in the canine jejunum at all three sampling points (Figure 2). No significant changes were observed at the phylum level. All five classes of *Proteobacteria *were identified (Figure 7), but they varied in their proportions and in their response to treatment (Figure 8).

*α-Proteobacteria *were detected in all 5 dogs on days 0 and 14, and in 4 dogs on day 28. This bacterial group was decreased in all dogs on day 14 and 28, mostly due to a decrease in *Sphingomonadaceae*, but this effect was not significant (p = 0.12; Figure 8).

Individual differences were observed for *β-Proteobacteria *with *Alcaligenaceae, Burkholderiaceae*, and *Neisseriaceae *being the most abundant representatives (Table 2). For *Neisseria *spp. there was a moderate increase on day 14 and a decrease on day 28, but overall these changes were not significant (means: 0.24% on day 0, 0.37% on day 14, and 0.08% on day 28; p = 0.12). *δ-Proteobacteria *were observed in low abundance and no obvious changes were observed.

**Table 2:** Distributions of bacterial groups on the family level.

	**% of sequences**			**number of dogs (n = 5)**		
		
**Family**	**day 0**	**day 14**	**day 28**	**day 0**	**day 14**	**day 28**
	
Actinomycetaceae	1.64	0.43	0.29	4	4	5
Aerococcaceae	1.75	0.45	0.43	4	5	3
Alcaligenaceae	0.11	0.08	0.00	2	2	0
Bacteroidaceae	1.70	0.07	0.43	3	3	2
Burkholderiaceae	0.26	0.41	0.00	1	3	0
Campylobacteraceae	0.13	0.19	0.02	3	1	1
Cardiobacteriaceae	0.27	0.55	0.01	3	2	1
Carnobacteriaceae	0.72	0.03	0.01	3	2	2
Clostridiaceae	5.47	19.46	10.72	4	5	5
Clostridiales Family XI. Incertae Sedis	1.07	0.53	0.11	4	3	4
Comamonadaceae	0.66	0.17	0.09	3	4	2
Coriobacteriaceae	0.12	0.00	0.47	2	0	1
Corynebacteriaceae	7.02	13.33	1.30	4	5	5
Deinococcaceae	0.00	0.02	0.02	0	1	2
Dermabacteraceae	1.44	0.22	0.16	4	3	3
Desulfobulbaceae	0.02	0.02	0.00	1	1	0
Desulfomicrobiaceae	0.03	0.01	0.21	1	1	2
Dietziaceae	0.10	0.71	0.00	4	4	0
Enterobacteriaceae	4.65	3.64	52.66	5	5	5
Enterococcaceae	0.03	0.43	0.02	3	5	2
Erysipelotrichaceae	0.03	0.00	0.22	3	0	2
Eubacteriaceae	0.22	0.10	0.11	4	3	1
Flavobacteriaceae	0.28	7.55	0.15	4	4	5
Flexibacteraceae	0.01	0.23	0.04	1	1	1
Fusobacteriaceae	5.39	0.48	6.30	3	4	3
Geobacteraceae	0.18	0.02	0.01	3	1	1
Helicobacteraceae	0.57	0.04	0.00	3	1	0
Lachnospiraceae	0.11	0.04	0.03	3	3	2
Microbacteriaceae	0.29	0.11	0.05	3	3	2
Micrococcaceae	0.18	0.03	0.01	3	3	1
Moraxellaceae	33.66	23.23	18.42	4	5	5
Mycoplasmataceae	0.03	0.00	0.22	1	0	2
Neisseriaceae	0.34	0.52	0.10	4	4	2
Nocardiaceae	0.00	0.11	0.07	0	3	2
Nocardioidaceae	0.04	0.00	0.02	3	0	1
Pasteurellaceae	0.72	17.95	0.74	4	5	5
Peptococcaceae	0.48	0.00	0.03	3	0	3
Peptostreptococcaceae	0.39	0.05	0.04	4	1	2
Porphyromonadaceae	1.57	0.01	1.12	4	1	4
Prevotellaceae	2.09	0.04	0.00	3	2	0
Propionibacteriaceae	0.15	0.80	0.06	4	5	2
Pseudonocardiaceae	0.00	0.11	0.00	0	3	0
Rhizobiaceae	0.00	0.17	0.01	0	3	1
Rhodobacteraceae	0.05	0.25	0.07	2	2	1
Ruminococcaceae	0.72	0.00	0.39	3	1	3
Sphingomonadaceae	3.38	0.00	0.07	3	0	2
Spirochaetaceae	14.15	0.02	0.37	5	2	3
Staphylococcaceae	0.14	0.06	0.14	2	3	4
Streptococcaceae	1.85	1.25	0.76	5	4	5
Streptomycetaceae	0.22	0.00	0.00	3	0	0
Succinivibrionaceae	0.16	0.00	0.29	1	0	3
Thermomicrobiaceae	0.02	0.01	0.01	2	1	1
Veillonellaceae	0.72	0.47	0.72	4	4	3
Xanthomonadaceae	0.66	1.32	0.06	4	4	3
other	4.02	4.27	2.42	n/a	n/a	n/a

The table shows the percentages of total sequences and the number of dogs that harbored those taxa at the 3 treatment periods. (day 0 = baseline; day 14 = after 14 days of tylosin administration; day 28 = 2 weeks after cessation of tylosin therapy).

*γ-Proteobacteria *were the most predominant group and were identified in all 5 dogs at all time points. Sequences of *Escherichia coli-*like organisms increased significantly by day 28 (p = 0.04) (Figure 3). This increase was observed in 3 dogs, where *Escherichia coli-*like organisms became the predominant group by day 28. *Pasteurella *spp. increased significantly (Table 2) by day 14, and returned to baseline values on day 28 (p = 0.04). This increase on day 14 was observed in 4 out of 5 dogs (Figure 2). *Moraxallaceae *decreased in 4 of 5 dogs on day 14, but increased in the remaining dog (Table 2, Figure 6).

A significant change was observed for ε-*Proteobacteria *(Figure 8; p = 0.039). Sequences belonging to this class were observed in 5 dogs on day 0, but only in 1 dog each on days 14 and 28 (p = 0.013). Decreases in *Helicobacteariaceae *and *Campylobacteriaceae *were both contributing to this change in ε-*Proteobacteria *(Table 2).

### Actinobacteria

Sequences belonging to the phylum *Actinobacteria *were identified in all dogs at all time points. No consistent changes in response to tylosin were observed on the phylum level. However, significant changes were observed for some bacterial taxa within this phylum. *Dietziaceae *increased significantly by day 14 (Figure 6; p = 0.03). This group increased in 3 dogs, remained stable in 1 dog, and was not detected in the remaining dog. Interestingly, no sequences belonging to *Dietziaceae *were detectable on day 28. *Streptomycetaceae *were detected in 3 dogs on day 0, but in none of the dogs on days 14 or 28 (Table 2; p = 0.039). *Actinomycetaceae *decreased in 4 of 5 dogs, but increased in the remaining dog on day 14. No *Bifidobacterium *spp. were detected in any of the samples.

## Discussion

Assessment of microbial diversity in the small intestine of dogs remains challenging, because anesthesia is required to obtain a sample, followed by either endoscopic or surgical collection of intestinal samples. Anesthesia may alter intestinal motility, and also repeated endoscopy may lead to perturbations of the intestinal microbiota. Therefore, the response of the jejunal microbiota to tylosin was evaluated in healthy Beagle Dogs each with a pre-existing jejunal fistula [21]. All dogs were accustomed to their fistula for several years and it is, therefore, unlikely that the presence of this fistula has impacted the intestinal microbiota. We collected samples using a sterile cytology brush that was advanced through the fistula. This approach is easier, faster, and more reproducible compared to the aspiration of jejunal content. Furthermore, because an endoscope is too large to advance through the small lumen of the fistula, intestinal biopsies would have to be collected in a blinded fashion, which might have increased the variation in the sampling procedure. In contrast, mucosal brushings are technically easier to obtain and have been shown to be highly reproducible [22]. We speculate that mucosal brushings represent a mixture of luminal content and the mucosa-adherent microbiota [23].

In this study, massive parallel 16S rRNA gene pyrosequencing proved to be a powerful and sensitive method for the further characterization of canine small intestinal microbiota. In previous studies using a comparative 16S rRNA gene based approach, up to 6 different bacterial phyla have been identified in the canine intestine [2,24] We have identified 4 additional phyla that were not previously reported in dogs: *Tenericutes, Cyanobacteria, Verrucomicrobia*, and *Chloroflexi*. These bacterial phyla were present at low abundance, with less than 1% of all pyrosequencing tags. The ecological significance of these low abundant bacterial phyla in the canine intestine remains to be determined. Furthermore, due to their low abundance, it was not possible to appreciate any significant effect due to tylosin treatment. While the overall composition of the small intestinal microbiota on a phylum through genus level was similar as reported previously in the canine duodenum using 16S rRNA gene analysis [2,24], the pyrosequencing approach has revealed a much higher richness on a species and strain level (Table 1). Rarefaction curves (Figure 1) revealed that with the number of here obtained sequencing tags per sample (mean ± SD: 3188 ± 1091), we have underestimated the number of OTUs at 1% dissimilarity, but obtained a reasonable coverage at 3% and 5% dissimilarity. Our calculations revealed that the canine jejunum harbors between 32 and 666 (mean: 293) bacterial species and between 183 and 1,789 (mean: 950) bacterial strains. Approximately 38,000 sequence tags would need to be analyzed per jejunal sample to cover 100% of the predicted maximum OTUs present in the canine jejunum. Therefore, future studies evaluating the small intestinal microbiota will need to employ larger sequencing datasets to characterize changes in low abundant bacterial groups.

By altering the intestinal microbiota, antibiotics can exhibit either a deleterious or a beneficial effect on gastrointestinal health. In humans with antibiotic associated diarrhea, a disruption of the intestinal ecosystem may predispose to an overgrowth of pathogenic species (e.g., *C. difficile*) [25]. However, antimicrobials can also be useful in the treatment of intestinal disorders. The macrolide antibiotic tylosin is commonly used for the treatment of dogs with chronic diarrhea, but the exact mode of action of tylosin remains unclear [11,12]. Most dogs respond favourably within 3-5 days, and stool consistency remains normal during treatment. However, diarrhea often reappears within weeks after discontinuation of administration [12]. Tylosin belongs to the macrolide class of antibiotics that is characterized by a multi-membered lactone ring [26]. Antibiotics of the macrolide class inhibit bacterial protein synthesis by binding to the L27 protein of the 50S ribosomal subunit. This inhibits the translocation of peptidyl-tRNA from the acceptor to the donor side on the ribosome, as well as the initial steps of assembly of the 50S subunit [26]. Macrolides are more effective in crossing the cell membrane of gram-positive bacteria compared to gram-negatives [27]. Therefore, the proposed antibiotic activity of tylosin is directed against gram-positive bacteria (e.g., *Stapylococcus *spp., *Streptococcus *spp., and *Clostridium *spp.) and also against some *Mycoplasma *and *Chlamydia *spp. While tylosin also has an effect against some gram-negative bacteria (e.g., *Campylobacter *spp., *Helicobacter pylori*, and *Pasteurella *spp.) it has no apparent effect against members of the *Enterobacteriaceae *(e.g., *Escherichia coli*) [27].

Antibiotics might exhibit their anti-diarrheal effect by either reducing total bacterial load in the gut or by modulating the proportions of specific bacterial taxa and, therefore, altering bacterial metabolites that affect the gastrointestinal tract. The here used pyrosequencing approach does not allow us to draw conclusions about changes in total bacteria within the intestine, as we did not include any measure for total bacterial load in our mucosal brushing samples. However, our approach shows changes in relative proportions of specific bacterial taxa in response to tylosin in a more comprehensive fashion than previously reported [9,18]. Recent studies in humans have evaluated the response of intestinal microbiota to a short-course treatment with amoxicillin or ciprofloxacin on fecal microbiota [8,16]. Similar to our results, antibiotic treatment led to major shifts in the dominant fecal bacterial populations, starting within 24 hours of administration [16]. Furthermore, ciprofloxacin affected the abundance of approximately one third of all bacterial taxa [8]. The human fecal microbiota proved to be generally resilient, and most taxa returned to baseline within 30 days, but some bacterial taxa failed to recover for up to 6 months [8,16].

In this study evaluating the small intestinal microbiota, we observed significant changes in the canine small intestinal microbiota in response to tylosin. Results of the Unifrac distance metric indicated that the jejunal microbiota of individual dogs were phylogentically more similar during tylosin administration. Samples tended to cluster during tylosin administration, indicating that such changes were due to treatment effect rather than temporal variation. Furthermore, in previous studies, using either bacterial culture or DGGE analysis, it has been shown that the major bacterial groups in the canine jejunum display temporal stability over time [22,28], further suggesting that the observed changes were indeed caused by tylosin treatment.

In general, the observed microbial shifts occurred in three major patterns: (a) bacterial groups that decreased in their proportions by day 14 and rebounded by day 28, (b) bacterial groups that decreased in their proportions by day 14 and failed to recover by day 28, and (c) bacterial groups that increased in their proportions by day 14 and returned to baseline values by day 28. We also observed unexpected highly individualized responses to tylosin treatment for specific bacterial taxa in some dogs. For dogs with diarrhea it is currently unknown if the effect of tylosin is mediated by a reduction in total bacterial load, by suppression of a single pathogen, or by an immunomodulatory effect [12]. Our findings show that tylosin affects the proportion of various bacterial groups in the intestine. It is, therefore, unlikely that tylosin would have solely an effect on a single pathogen in clinical cases. It can be hypothesized that some of the observed shifts in microbial populations might contribute to the beneficial effect observed in dogs with chronic enteropathies. Examples of the beneficial effect of antibiotics may include altered concentrations of secreted metabolic products, decreased competition for nutrients or vitamins, altered cross-talk with the intestinal immune system, or a modification of cellular metabolism [29-31]. To prove this hypothesis, evaluation of these bacterial groups in clinical studies involving diseased animals are required. Furthermore, changes in bacterial populations will need to be correlated with treatment outcome.

It is interesting that the proportions of *Enterococcus*-like organisms, which are commonly used in probiotic formulations increased significantly during tylosin treatment. *Enterococcus *spp. have been reported to be resistant to tylosin in several animal studies [17,32], and suppression of the commensal microbiota by antibiotic treatment may have allowed the proliferation of this bacterial group. For example, in one study using a continues flow culture model, a tylosin-resistant exogenous *E. faecium *strain could maintain itself only in the presence of tylosin [17]. These results support the concept that tylosin may promote the growth of potentially beneficial commensal bacteria such as *Enterococcus *spp., which may have probiotic characteristics. A similar concept has also been suggested for the effect for the antibiotic metronidazole, also commonly used for treatment of dogs with chronic enteropathies. In humans, metronidazole increased the proportions of *Bifidobacterium *spp. [33]. However, it remains unclear if a mere increase in the proportions of specific bacterial genera is sufficient to exhibit a probiotic effect. It is currently also unknown, if minor changes (i.e., less than 10-fold) as observed have any significant impact on intestinal health. To prove the concept that antibiotics may be able to promote proliferation of probiotic bacteria, it would be useful to isolate native *Enterococcus *strains and evaluate their functional interactions with other members of the intestinal microbiota and also evaluate their probiotic properties in dogs with gastrointestinal disease.

Tylosin is usually considered safe for long-term use in dogs [34]. However, in this study we observed some unexpected microbial shifts, which may suggest that tylosin, similar to other antibiotics, can lead to a disruption of the intestinal ecosystem and also have potentially deleterious effects on gastrointestinal health. We observed significant increases for *Pasteurella *spp., *E. coli-*like organisms, and a dramatic increase in *C. perfringens*-like organisms in one dog. Tylosin is prescribed for the therapy of upper respiratory infections associated with *Pasteurella multocida*. However, this group increased significantly during the treatment period. It remains unclear, if *Pasteurella multocida *has developed resistance to tylosin in the here studied dogs, or if the intestinal phylotypes differ from those isolated from the lung. Tylosin appears to be an appropriate antibiotic for the treatment of *C. perfringens*-associated diarrhea in canine patients, although resistant strains have been observed [10]. Similarly, in a chicken model of necrotizing enteritis, tylosin quantitatively decreased the proportion of mucolytic *C. perfringens *[18]. However in this study, the percentage of *C. perfringens*-like organisms increased from 21.8% on day 0 to 86.7% on day 14 in one dog, suggesting that this dog harbored a resistant strain. Our results also suggest that the proposed mode of action of an antibiotic on different bacterial genera does not necessarily match the *in vivo *effects, as several bacterial groups that are considered to be sensitive to tylosin increased in their proportions. Because of the nature of an ecosystem, the changes that are induced by an antibiotic on one set of organisms will affect others, and this is not necessarily predicted by *in vitro *antibiotic sensitivities.

*E. coli-*like organisms, a bacterial group that has also been associated with a negative impact on gastrointestinal health in dogs [24,35] increased significantly by day 28. The enrichment of *E. coli-*like organisms is not surprising, as this group is intrinsically resistant to tylosin, and similar increases have been observed in pigs after tylosin treatment [36]. However, we have no obvious explanation why this effect was observed on day 28 rather than day 14, the last day of tylosin administration. Also, based on the techniques used, it is not possible to determine if a bacterial population proliferated or simply increased in proportion because other bacteria were affected (directly or indirectly) by the antibiotic treatment.

While *E. coli-*like organisms and *C. perfringens *increased in some of the dogs, this was not associated with any obvious clinical signs of gastrointestinal disease. We speculate that despite obvious changes in microbial populations, the intestinal ecosystem has enough functional redundancy to maintain gastrointestinal health. Similar findings have also been reported in humans, where short-term courses of antibiotics led to significant shifts in fecal microbiota patterns, yet no obvious gastrointestinal signs were observed [8,16]. However, all these studies, including the present one, have evaluated healthy individuals, which may harbor a stable intestinal ecosystem that has enough functional redundancy to withstand short-term modulations. It is currently unknown how antibiotics affect dogs with gastrointestinal disease that may be more susceptible to such treatments. Of interest would be also to evaluate the long-term effects of antibiotics on the temporal stability of the intestinal microbiota and their influence on gastrointestinal health. It might be possible that the microbiota in animals undergoing a course of antibiotic treatment is less stable and, therefore, at an increased risk for gastrointestinal disease or infections. Follow up studies over a period of years would be needed to answer this question. In this study we have evaluated healthy dogs, and it is possible that tylosin has a different effect on the microbiota in dogs with signs of gastrointestinal disease. It is suspected that diseased dogs have an altered microbial composition, and it is possible that tylosin results in modulations in microbiota that differ from those observed in the here evaluated healthy animals. Evaluating endoscopically obtained pre- and post treatment samples from dogs with tylosin-responsive diarrhea would be valuable. Future studies will need also to evaluate intestinal contents for changes in bacterial metabolites or gene expression in response to antibiotic treatment as a measure of functional redundancy of the intestinal microbiota.

Studies in humans have shown that the fecal microbiota are generally resilient to short-term modulations by antibiotics, but pervasive effects might last for several months for specific bacterial taxa [8,16]. The resilience of a microbial community reflects its capability to return to baseline after disturbances to the community (i.e., antibiotic treatment) have ceased. Less is known about the resilience of the small intestinal microbiota. Our results illustrate the complexity of the intestinal microbiota and the challenges associated with evaluating the effect of antibiotic administration on the various bacterial groups and their potential interactions. Our results indicate that tylosin may lead to prolonged effects on the composition and diversity of jejunal microbiota. On day 28, the phylogenetic composition of the microbiota was similar to day 0 in only 2 of 5 dogs. Bacterial diversity as measured by the Shannon-Weaver diversity index resembled the pre-treatment state in 3 of 5 dogs. Several bacterial groups changed in their proportions in response to tylosin. After cessation of tylosin, the phyla *Firmicutes *and *Fusobacteria *tended to return to pretreatment values within 14 days. Other phyla, such as *Bacteroidetes*, *Proteobacteria*, and *Spirochaetes *did not return to their pre-treatment proportions. Tylosin had also a pervasive effect on several bacterial groups that failed to recover by day 28 (i.e., 14 days after tylosin therapy had been completed). Those groups included *Spirochaetes, Streptomycetaceae, Sphingomonadaceae*, and *Prevotellaceae*. Tylosin has a known activity against *Spirochaetes *[37]. *Spirochaetes *have been associated with intestinal disease in chickens and pigs, but their pathogenic role in dogs remains unclear, as they are commonly observed in healthy dogs as well as dogs with diarrhea [2,24,38]. The clinical significance of *Sphingomonadaceae, Prevotellaceae*, and *Streptomycetaceae *in the small intestine of dogs has, to our knowledge, not been evaluated to date. Furthermore, future studies with longer follow-up periods than 14 days after treatment cessation will be useful to evaluate the long-term effect of tylosin on the jejunal microbiota. Result of such studies may indicate the time needed for the microbiota to return to its pre-treatment state.

## Conclusion

In conclusion, using deep massive parallel pyrosequencing we identified additional bacterial phyla and demonstrated the enormous species richness present in the small intestine of healthy dogs. We have demonstrated a profound and pervasive effect of tylosin on microbial diversity and various bacterial groups. These bacterial groups may represent candidates for exploration in clinical studies, and their changes will need to be correlated with clinical outcome, to further understand the effect of tylosin on gastrointestinal health.

## Methods

### Animals

Five healthy dogs, each with a pre-existing jejunal fistula inserted approximately 60 cm distal to the pylorus were used in this study [21]. All dogs were considered healthy and had no recent history of gastrointestinal disease. All dogs were unrelated and approximately two years old. Their body weights ranged from 12 to 19 kg, and their body condition scores ranged between 3 and 4 (median 3) on a 5-point scale. The dogs received a commercial dry dog food (Mastery Adult Essential Maintenance, Dog'n Cat International, Vauvert, France) twice a day throughout the study period. According to the manufacturer, the food composition was 28% crude protein, 20% crude fat, 7% crude ash, and 2.5% crude fibre. During the study period, the dogs were cared for by the same personnel. All dogs were housed at the same laboratory animal unit at the Faculty of Veterinary Medicine, University of Helsinki, Finland. Dogs were housed in separate pens and treated individually. All dogs were fed at the same time each day.

Tylosin was administered at 20 to 22 mg/kg q 24 hr for a period of 14 consecutive days. This is the same dose that has previously been recommended for the treatment of tylosin-responsive diarrhea [34].

### Sample collection

The study had been approved by the Finnish Ethical Committee with license number ESLH-2007-09833/Ym-23. Mucosal brush samples were collected by advancing a sterile cytology brush through the fistula as described previously [23]. Samples were collected on day 0 (baseline), day 14 (after 14 days of tylosin administration), and day 28 (14 days after withdrawal of tylosin). To ensure consistency in sample collection, the same person collected all the samples during the whole study period. Furthermore, the samples were obtained according to a timetable with each sample collected exactly at the same time after feeding (i.e. dogs were fed consecutively, so that each sample could be collected in each dog at the same time after feeding). Samples were homogenized, properly labeled, and immediately frozen and stored at -80°C until further analysis.

### DNA extraction

Genomic DNA was extracted individually from all jejunal samples using a modified bead beating method followed by phenol:chloroform:iso-amylalcohol extraction as described previously for canine small intestinal brush samples [23].

### Massive parallel 16S rRNA gene pyrosequencing

Bacterial tag-encoded FLX amplicon pyrosequencing (bTEFAP) based upon the V4-V5 region of the 16S rRNA gene was performed as described previously [39] at the Research and Testing Laboratory (Lubbock, TX.).

### Sequence analysis

Following sequencing, all failed sequence reads, low quality sequence ends (Q20 based scores as determined by the Roche base calling algorithm) and tags were removed. Datasets were depleted of any non-bacterial ribosomal sequences and chimeras using custom software described previously [40] and the Black Box Chimera Check software B2C2 (Gontcharova et al 2009, in press, described and freely available at http://www.researchandtesting.com/B2C2.html). Sequences less than 150 bp were removed. To determine the identity of bacteria in the remaining sequences, sequences were first compared against a database of high confidence 16S rRNA gene sequences derived from NCBI using a distributed BLASTn .NET algorithm [41]. Database sequences were characterized as high quality based upon the criteria of RDP ver 9 [42]. Using a .NET and C# analysis pipeline, the resulting BLASTn outputs were compiled, validated using taxonomic distance methods when necessary (multiple hits with similar BLASTn statistics), and data reduction analysis was performed as described previously [20]. For distance method validation, the top 25 BLASTn hits were automatically extracted, trimmed and aligned using MUSCLE, a distance matrix formed using PHYLIP, and the hits ranked based upon distance scores and BLASTn statistics. Identifications were resolved based upon a preference for distance scoring. Rarefaction of 200 bp trimmed, non-ribosomal sequence depleted, chimera depleted, high quality reads was performed as described previously [20]. Based upon the BLASTn derived sequence identity (percentage of total length query sequence, which aligns with a given database sequence validated using distance methods), the bacteria were classified at the appropriate taxonomic levels based upon the following criteria: sequences with identity scores to known or well characterized 16S sequences greater than 97% were resolved at the species level, between 95% and 97% at the genus level, between 90% and 95% at the family level, and between 80% and 90% at the order level [19]. After individually resolving the sequences within each sample to its best hit, the results were compiled to provide relative abundance estimations at each taxonomic level. Evaluations presented at a given taxonomic level, except the species level, represent all sequences resolved to their primary genera identification or their closest relative (where indicated).

### Statistical analysis

To determine whether the bacterial communities were different between the treatment periods, the UniFrac distance metric was used [43]. This method measures the phylogenetic distance among bacterial communities in a phylogenetic tree [43], and provides a measure of similarity among communities in different samples. To compare the similarity of the jejunal microbiota in all dogs at the three time points, all the pair-wise distances between the communities were computed. To visualize the clustering of the samples along the first 3 axes of maximal variance, Principal Coordinate Analysis (PCA) was used. PCA allows visualization whether any environmental factors (i.e., tylosin treatment) would group the communities together (Figure 5).

Differences in bacterial groups between time points were determined using repeated measures ANOVA or Friedman's test where appropriate (Prism5, GraphPad Software Inc, San Diego, Calif). Fisher's exact tests were used to compare proportions of dogs that harbor specific bacterial taxa among time points. The data were used to calculate the Shannon-Weaver bacterial diversity index, which yields information about species diversity in bacterial communities. The Shannon-Weaver index (Hs) was defined as -∑p_*i*_ln(p_*i*_), where p_*i *_is the proportion of individual bacteria found in a certain species [44]. The Shannon-Weaver index takes into account the abundance and the evenness of the species present within a community. Microbial communities with higher species richness and an even distribution (i.e., each species is present in similar proportions) will have a higher Hs than communities with a lower species richness, or communities with high species richness but where a few species predominate. To estimate the total number of OTUs present in each sample, the coverage-based nonparametric richness estimators Ace and Chao 1 were calculated. Rarefaction curves were produced using the software program DOTUR [45]. Rarefaction analysis is used to estimate diversity and can serve as an indicator for the completeness of sampling [46]. To predict the maximum number of OTUs present in the canine jejunum, a Richards equation [47] was fit to the rarefaction curves [20]. The Richards equation has parameters C1 and C2 with the equation C1 = A × (1+(B - 1) × EXP (-C × ((C2) - D)))^(1/(1-B))^, where C1 is the OTU estimated and C2 is the number of sequences sampled [20].

## Abbreviations

16S rRNA: 16S ribosomal RNA; PCR: polymerase chain reaction; SIBO: small intestinal bacterial overgrowth; ARD: antibiotic responsive diarrhea; TRD: tylosin responsive diarrhea; DGGE: denaturing gel gradient electrophoresis; bTEFAP: bacterial tag-encoded FLX amplicon pyrosequencing; ANOVA: analysis of variance; PCA: Principal Component Analysis; OTU: operative taxonomical unit

## Authors' contributions

JSS, EW, JH, and TS conceived the study design; JH and EW performed sample collection; SED performed pyrosequencing analysis; JSS, SED, and JMS performed statistical analysis, and all authors contributed to the writing of the manuscript.
